# PKM2-dependent glycolysis promotes NLRP3 and AIM2 inflammasome activation

**DOI:** 10.1038/ncomms13280

**Published:** 2016-10-25

**Authors:** Min Xie, Yan Yu, Rui Kang, Shan Zhu, Liangchun Yang, Ling Zeng, Xiaofang Sun, Minghua Yang, Timothy R. Billiar, Haichao Wang, Lizhi Cao, Jianxin Jiang, Daolin Tang

**Affiliations:** 1 Department of Pediatrics, Xiangya Hospital, Central South University, Changsha, Hunan 410008, China; 2 Department of Surgery, University of Pittsburgh, Pittsburgh, Pennsylvania 15219, USA; 3 Center of DAMP Biology, The Third Affiliated Hospital of Guangzhou Medical University, Guangzhou, Guangdong 510510, China; 4 State Key Laboratory of Trauma, Burns and Combined Injury, Research Institute of Surgery, Research institute for Traffic Medicine of People's Liberation Army, Daping Hospital, Third Military Medical University, Chongqing 400042, China; 5 Laboratory of Emergency Medicine, The Feinstein Institute for Medical Research, Manhasset, New York 11030, USA

## Abstract

Sepsis, severe sepsis and septic shock are the main cause of mortality in non-cardiac intensive care units. Immunometabolism has been linked to sepsis; however, the precise mechanism by which metabolic reprogramming regulates the inflammatory response is unclear. Here we show that aerobic glycolysis contributes to sepsis by modulating inflammasome activation in macrophages. PKM2-mediated glycolysis promotes inflammasome activation by modulating EIF2AK2 phosphorylation in macrophages. Pharmacological and genetic inhibition of PKM2 or EIF2AK2 attenuates NLRP3 and AIM2 inflammasomes activation, and consequently suppresses the release of IL-1β, IL-18 and HMGB1 by macrophages. Pharmacological inhibition of the PKM2–EIF2AK2 pathway protects mice from lethal endotoxemia and polymicrobial sepsis. Moreover, conditional knockout of PKM2 in myeloid cells protects mice from septic death induced by NLRP3 and AIM2 inflammasome activation. These findings define an important role of PKM2 in immunometabolism and guide future development of therapeutic strategies to treat sepsis.

## Abstract (web summary)

Inflammation involves a Warburg effect that switches cellular metabolism to glycolysis. Here the authors show this switch drives IL-1β, IL-18 and HMGB1 release from macrophages by activating the NLRP3 and AIM2 inflammasomes via protein kinase R phosphorylation, a pathway that can be inhibited to prevent sepsis in mice.

## Graphical abstract

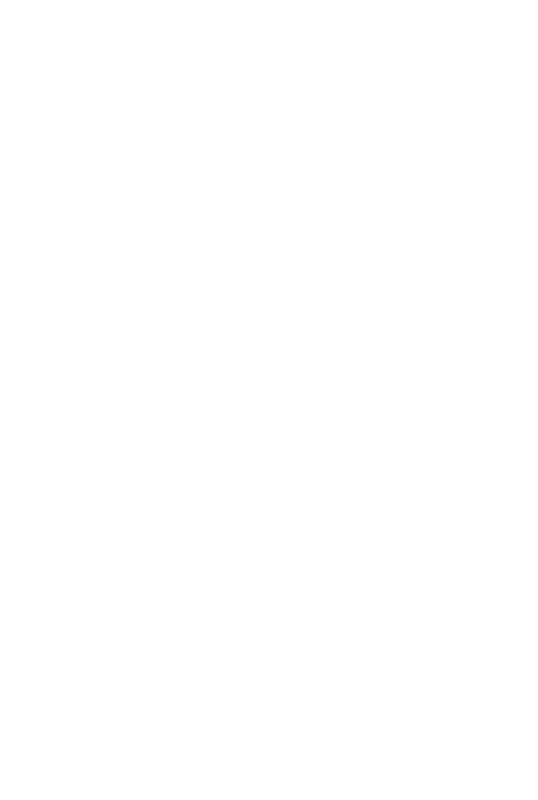

Despite the implementation of goal-directed care (fluid resuscitation, antibiotics, source control and vasopressors), severe sepsis and septic shock are the most common cause of death in intensive care units. An excessive host response associated with a non-resolving systemic inflammatory response syndrome contributes to the pathogenesis of sepsis. Among the common bacterial causes of sepsis are Gram-negative bacilli. A major component of Gram-negative bacteria, lipopolysaccharide (LPS), induces the secretion and release of multiple proinflammatory mediators such as tumour necrosis factor (TNF), interleukin (IL)-1β and high mobility group box 1 (HMGB1). In contrast to early proinflammatory cytokines (for example, TNF and IL-1β), HMGB1 is released in a delayed manner by LPS-stimulated macrophages^1^. Macrophages can efficiently release HMGB1, particularly when the initial LPS priming is accompanied by a second stimulus such as adenosine triphosphate (ATP)^2^. Once released, HMGB1 binds to cell-surface receptors (for example, toll-like receptors and receptor for advanced glycation end products) and propagates the inflammatory response. Administration of anti-HMGB1 antibodies and inhibitors (for example, ethyl pyruvate, nicotine and chloroquine) protects mice against lethal experimental sepsis^3^, establishing HMGB1 as a potential therapeutic target for sepsis and other inflammatory diseases^4^.

The inflammasome pathways contribute to the inflammatory response in sepsis^5^. Inflammasomes are protein complexes assembled on recognition of exogenous and endogenous danger signals and serve as platforms for activation of canonical caspase-1 or non-canonical caspase-11 and secretion of proinflammatory cytokines (for example, IL-1β, IL-18 and HMGB1) to engage immune and inflammatory responses^6^. In particular, phosphorylation and activation of the eukaryotic translation initiation factor 2 alpha kinase 2 (EIF2AK2, also termed PKR) is required for inflammasome-dependent IL-1β and HMGB1 release by macrophages^7^. However, the precise molecular mechanism underlying the regulation of EIF2AK2 phosphorylation in sepsis is not well understood.

Glycolysis is the metabolic pathway that converts glucose into pyruvate. Pyruvate can be used in either anaerobic respiration if no oxygen is available or in aerobic respiration via the tricarboxylic acid cycle, which yields much more usable energy for the cell. Aerobic glycolysis is controlled by various glycolytic enzymes. Lactate dehydrogenase (LDH) converts pyruvate to lactate when oxygen is absent or in short supply. The M2 isoform of pyruvate kinase, muscle (PKM2), catalyses the final and also a rate-limiting reaction in the glycolytic pathway. PKM2 is present in few types of proliferating normal cells, but is present at high levels in cancer cells and activated immune cells. PKM2-dependent aerobic glycolysis promotes IL-1β and HMGB1 release in LPS-stimulated macrophages^8^,^9^. However, whether PKM2-dependent glycolysis regulates IL-1β and HMGB1 release by regulating inflammasome activation in macrophages is unknown.

Here we provide the first evidence that upregulation of PKM2-dependent glycolysis contributes to IL-1β, IL-18 and HMGB1 release by selective activation of EIF2AK2-dependent NLR family, pyrin domain containing 3 (NLRP3) and absent in melanoma 2 (AIM2) inflammasome in macrophages. Pharmacological and genetic inhibition of the PKM2–EIF2AK2 pathway attenuates activation of NLRP3 and AIM2 inflammasomes and limits the release of IL-1β, IL-18 and HMGB1 *in vitro* or *in vivo*. These findings improve our understanding of the emerging role of immunometabolism in inflammation and will guide future development of therapeutic strategies to treat sepsis.

## Results

### PKM2 inhibitor blocks inflammasome activation

**Figure 1:**
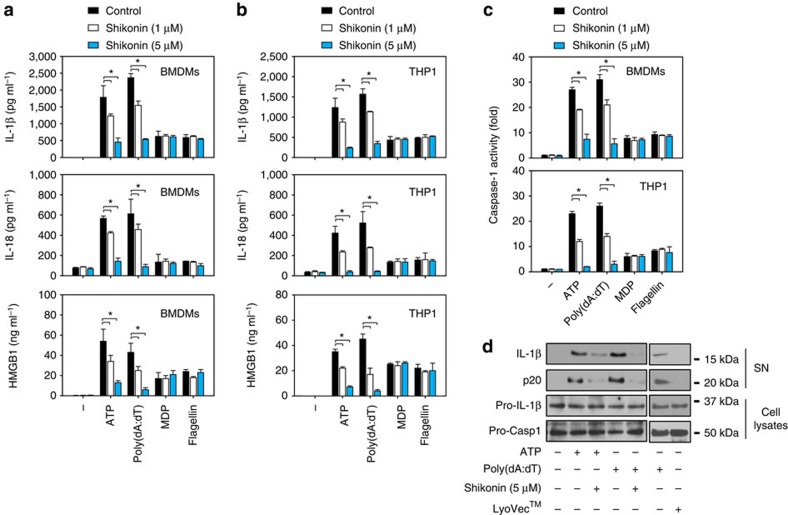
Pharmacological inhibition of PKM2 impairs inflammasome activation. LPS (500 ng ml^*−*1^, 3 h)-primed mouse BMDMs and human PMA-differentiated THP1 cells were respectively treated with inflammasome activators (ATP (5 mM, 30 min), poly(dA:dT) (1 μg ml^*−*1^, 8 h), MDP (200 ng ml^*−*1^, 8 h) or flagellin (200 ng ml^*−*1^, 8 h)) in the absence or presence of shikonin at the same time (1 and 5 μM). IL-1β, IL-18 and HMGB1 (**a**,**b**) in supernatants and caspase-1 activity (**c**) in whole-cell extract were assayed with ELISA or activity assay kit (*n*=3, *P<0.05, ANOVA LSD test). In parallel, the levels of IL-1β and cleaved caspase-1 (p20) in culture supernatants (SN) and the precursors of IL-1β (pro-IL-1β) and caspase-1 (pro-caspase-1) in lysates of BMDMs were assayed using western blot (**d**). As a control, the cationic lipid transfection reagent LyoVec did not induce the cleavage of IL-1β and caspase-1 in BMDMs. All quantification data expressed as means±s.e.m of three independent experiments. Western blot data are representative of two independent experiments.

Shikonin is a potent PKM2 inhibitor in cancer cells and macrophages^9^. To investigate the function of PKM2 in inflammasome activation, we first treated LPS-primed mouse bone marrow-derived macrophages (BMDMs) with inflammasome inducers in the presence and absence of shikonin. Shikonin dose-dependently inhibited IL-1β, IL-18 and HMGB1 release in activated BMDMs following treatment with NLRP3 inflammasome activator (for example, ATP) or AIM2 inflammasome activator (for example, poly(dA:dT)) (Fig. 1a). In contrast, shikonin did not affect the release of IL-1β, IL-18 and HMGB1 that was induced by NLR family, pyrin domain containing 1 (NLRP1) inflammasome activators (for example, muramyl dipeptide (MDP)) or NLR family, CARD domain containing 4 (NLRC4) inflammasome activators (for example, flagellin) (Fig. 1a). Similarly, shikonin also dose-dependently inhibited the release of IL-1β, IL-18 and HMGB1 in phorbol 12-myristate 13-acetate (PMA)-differentiated THP1 cells and mouse peritoneal macrophages (PMs) following stimulation with ATP or poly(dA:dT), but not MDP or flagellin (Fig. 1b and Supplementary Fig. 1). Given the important role of caspase-1 in canonical inflammasome-mediated secretion of proinflammatory cytokines, we next examined the effect of PKM2 inhibition on caspase-1 activation using a fluorometric activity assay kit that recognizes the sequence YVAD. In LPS-primed BMDMs, PMA-differentiated THP1, and PMs, shikonin significantly inhibited caspase-1 activation triggered by stimulation with ATP or poly(dA:dT), but not MDP or flagellin (Fig. 1c and Supplementary Fig. 1). Similarly, western blot analysis demonstrated that shikonin reduced extracellular levels of IL-1β and cleaved caspase-1 (p20) in the culture supernatants of BMDMs primed by LPS and subsequently stimulated with ATP or poly(dA:dT) (Fig. 1d). In contrast, LyoVec (the transfection reagent) did not affect caspase-1 activation (Fig. 1d). Collectively, these findings suggest that pharmacologic inhibition of PKM2 by shikonin selectively suppresses NLRP3 and AIM2 inflammasome activation.

### Knockdown of PKM2 blocks inflammasome activation

**Figure 2:**
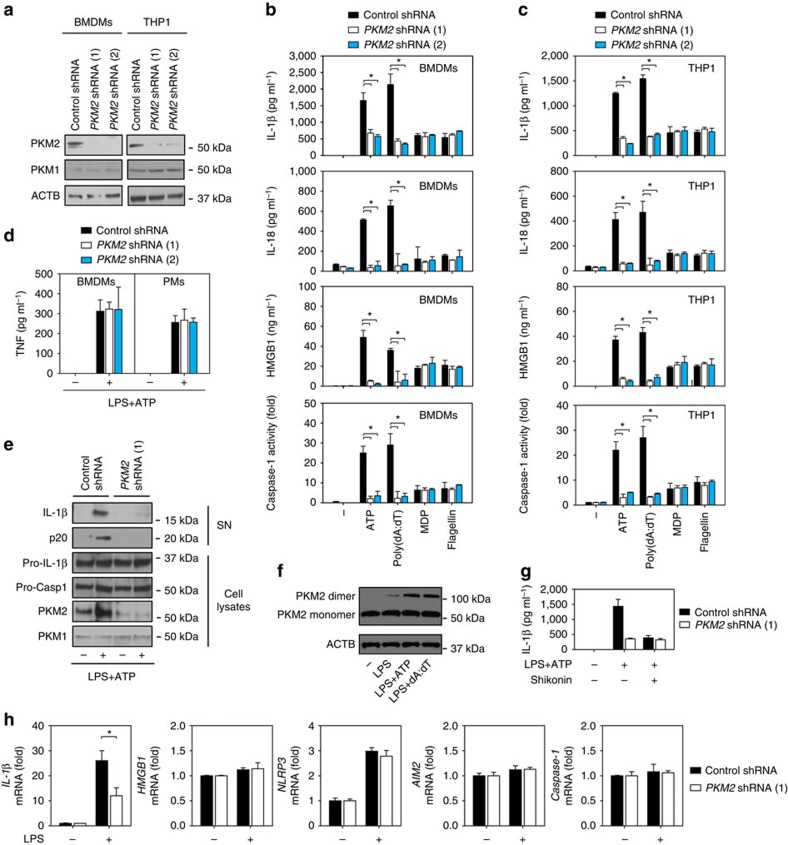
Genetic inhibition of *PKM2* suppresses inflammasome activation. (**a**) Western blot analysis of PKM2 expression in BMDMs and PMA-differentiated THP1 cells after knockdown of *PKM2* by specific shRNA for 48 h. (**b**,**c**) LPS (500 ng ml^*−*1^, 3 h)-primed BMDMs (**b**) and PMA-differentiated THP1 cells (**c**) were treated with various inflammasome activators (ATP (5 mM, 30 min), poly(dA:dT) (1 μg ml^*−*1^, 8 h), MDP (200 ng ml^*−*1^, 8 h) or flagellin (200 ng ml^*−*1^, 8 h)). Extracellular levels of IL-1β, IL-18 and HMGB1 and cellular levels of caspase-1 were assayed using ELISA or activity assay kit (*n*=3, *P<0.05, ANOVA LSD test). (**d**,**e**) In parallel, TNF levels were assayed with ELISA (**d**); IL-1β and cleaved caspase-1 (p20) in culture supernatants (SN) and the precursors of IL-1β (pro-IL-1β) and caspase-1 (pro-caspase-1) in lysates of BMDMs were assayed using western blot (**e**). (**f**) Native gel electrophoresis was performed using whole-cell extracts from LPS treatment alone (500 ng ml^*−*1^, 3 h) or LPS (500 ng ml^*−*1^, 3 h)-primed BMDMs after treatment with ATP (5 mM, 30 min) or poly(dA:dT) (1 μg ml^*−*1^, 8 h). (**g**) Shikonin (5 μM) did not increase *PKM2*-shRNA-mediated inhibition of IL-1β release in LPS (500 ng ml^*−*1^, 3 h)-primed BMDMs following treatment with ATP (5 mM, 30 min). (**h**) Q-PCR analysis of gene expression in indicated BMDMs following treatment with LPS (500 ng ml^*−*1^) for 3 h (*n*=3, *P<0.05, *t*-test). All quantification data expressed as means±s.e.m of three independent experiments. Western blot data are representative of two independent experiments.

To further confirm the role of PKM2 in the regulation of inflammasome activation, we suppressed PKM2 expression by lentiviral transduction with *PKM2*-specific shRNA. Knockdown of *PKM2* by shRNA (Fig. 2a) significantly impaired IL-1β, IL-18 and HMGB1 release by BMDMs (Fig. 2b), PMA-differentiated THP1 (Fig. 2c), and PMs (Supplementary Fig. 2) following stimulation with ATP or poly(dA:dT), but not MDP or flagellin. In contrast, the knockdown of *PKM2* in BMDMs and PMs did not affect LPS/ATP-induced release of other cytokines (for example, TNF) (Fig. 2d). Furthermore, the knockdown of *PKM2* also led to the inhibition of caspase-1 activation in BMDMs (Fig. 2b), PMA-differentiated THP1 (Fig. 2c), and PMs (Supplementary Fig. 2) following treatment with ATP and poly(dA:dT), but not MDP and flagellin. Similarly, western blot analysis demonstrated that knockdown of *PKM2* resulted in reduction of extracellular levels of IL-1β and cleaved caspase-1 (p20) in the culture supernatants of LPS/ATP-stimulated BMDMs (Fig. 2e). The Warburg effect in macrophages is due to dimeric or monomeric PKM2 (ref. 8). As expected, the dimeric PKM2 was increased in LPS treatment alone or LPS-primed BMDMs following treatment with NLRP3 inflammasome activator (for example, ATP) or AIM2 inflammasome activator (for example, poly(dA:dT)) (Fig. 2f). The PKM2 inhibitor shikonin did not increase *PKM2*-shRNA-mediated inhibition of IL-1β release in BMDMs following treatment with LPS/ATP (Fig. 2g). These findings suggest that genetic inhibition of *PKM2* by RNAi selectively suppresses NLRP3 and AIM2 inflammasome activation.

In addition to regulating inflammasome-mediated IL-1β maturation and secretion, we and others previously demonstrated that activation of PKM2 contributes to LPS-induced *IL-1β* (but not *HMGB1*) mRNA expression in macrophages^8^,^9^. Consistently, suppression of *PKM2* expression by shRNA inhibited LPS-induced *IL-1β* (but not *HMGB1*) mRNA expression in BMDMs (Fig. 2h). However, the protein level of pro-IL-1β was not significantly affected by PKM2 in BMDMs following treatment with LPS/ATP (Fig. 2e). The mRNA levels of *NLRP3*, *AIM2,* and *caspase-1* were not changed after knockdown of *PKM2* in BMDMs with or without LPS treatment (Fig. 2h). These findings suggest that PKM2 regulates activation of NLRP3 and AIM2 inflammasome, but not their mRNA expression.

### Glycolysis contributes to inflammasome activation

**Figure 3:**
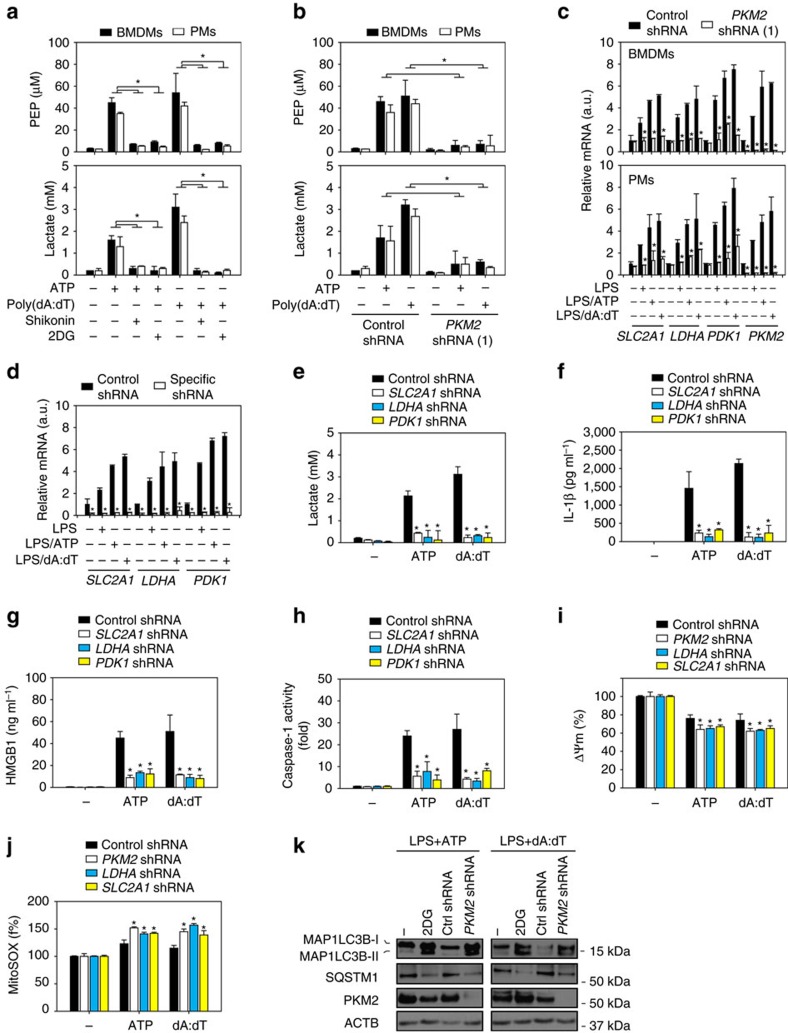
PKM2-dependent glycolysis is required for inflammasome activation. (**a**) LPS (500 ng ml^*−*1^, 3 h)-primed BMDMs and PMs were treated with inflammasome activators (ATP (5 mM, 30 min) or poly(dA:dT) (1 μg ml^*−*1^, 8 h)) in the absence or presence of shikonin (5 μM) or 2DG (2 mM). PEP and lactate levels were assayed using a commercial kit (*n*=3, *P<0.05). (**b**,**c**) Knockdown of PKM2 by shRNA prevented ATP (5 mM, 30 min) and poly(dA:dT) (1 μg ml^*−*1^, 8 h)-induced increases in PEP and lactate levels (**b**), as well as mRNA expression of glycolysis-related genes (**c**) in LPS (500 ng ml^*−*1^, 3 h)-primed BMDMs and PMs (*n*=3, *P<0.05 versus control shRNA group, ANOVA LSD test). In parallel, mRNA expression of indicated genes was assayed in BMDMs and PMs following treatment with LPS (500 ng ml^*−*1^, 3 h) alone (**c**). (**d**–**h**) Knockdown of *SLC2A1*, *LDHA* or *PDK1* by shRNA (**d**) inhibited ATP-(5 mM, 30 min) or poly(dA:dT) (1 μg ml^*−*1^, 8 h)-induced lactate production (**e**), IL-1β (**f**) and HMGB1 (**g**) release and caspase-1 activation (**h**) in LPS (500 ng ml^*−*1^, 3 h)-primed BMDMs (*n*=3, *P<0.05 versus control shRNA group, ANOVA LSD test). In parallel, mRNA expression of indicated genes was assayed in BMDMs following treatment with LPS (500 ng ml^*−*1^, 3 h) alone (**d**). (**i**,**j**) Knockdown of *PKM2*, *LDHA* or *SLC2A1* by shRNA increased mitochondrial membrane potential (ΔΨm) loss (**i**) and mitochondrial ROS production (MitoSOX) (**j**) with or without ATP (5 mM, 30 min) and poly(dA:dT) (1 μg ml^*−*1^, 8 h) treatment in LPS (500 ng ml^*−*1^, 3 h)-primed BMDMs (*n*=3, *P<0.05 versus control shRNA group, ANOVA LSD test). (**k**) Western blot analysis of MAP1LC3B-II and SQSTM1 in indicated LPS-primed BMDMs after treatment with ATP (5 mM, 30 min) and poly(dA:dT) (1 μg ml^*−*1^, 8 h). All quantification data expressed as means±s.e.m of three independent experiments. Western blot data are representative of two independent experiments.

Reasoning that PKM2 catalyses the final and also a rate-limiting reaction of the glycolytic pathway, we first analysed the levels of glycolytic metabolites (for example, phosphoenolpyruvate (PEP) and lactate) in response to NLRP3 or AIM2 inflammasome activator with or without the PKM2 inhibitor shikonin. Indeed, both shikonin and the glycolysis inhibitor 2-deoxy-D-glucose (2DG) inhibited increases in PEP and lactate levels in LPS-primed BMDMs and PMs following treatment with ATP or poly(dA:dT) (Fig. 3a). Importantly, the suppressed *PKM2* expression by shRNA limited the production of PEP and lactate in LPS-primed BMDMs and PMs following treatment with ATP and poly(dA:dT) (Fig. 3b). PKM2 has been reported to function in the nucleus to promote glycolysis by regulating the transcription of glycolysis-related genes such as solute carrier family 2 member 1 (*SLC2A1*, also termed *GLUT1*, which increases glucose uptake), lactate dehydrogenase A (*LDHA*, which increases lactate production) and pyruvate dehydrogenase kinase 1 (*PDK1*, which blocks the entry of pyruvate into the tricarboxylic acid cycle)^10^. The mRNA levels of *SLC2A1*, *LDHA* and *PDK1* were all increased in BMDMs initially by LPS alone or primed by LPS and subsequently stimulated with ATP or poly(dA:dT) (Fig. 3c). In contrast, knockdown of *PKM2* by shRNA also led to reduced mRNA expression of *SLC2A1*, *LDHA* and *PDK1* in macrophages stimulated with NLRP3 or AIM2 inflammasome activators (Fig. 3c). To determine whether PKM2-dependent glycolysis-related gene expression is required for the production of lactate in inflammasome activation, we suppressed expression of *SLC2A1*, *LDHA* or *PDK1* by specific shRNA targeting each enzyme in BMDMs (Fig. 3d). Knockdown of *SLC2A1*, *LDHA* or *PDK1* prevented increases in lactate levels (Fig. 3e), as well as IL-1β (Fig. 3f) and HMGB1 (Fig. 3g) release and caspase-1 activation (Fig. 3h) in response to ATP or poly(dA:dT). These findings suggest that PKM2-dependent glycolysis promotes NLRP3 and AIM2 inflammasome activation in macrophages.

Given that glycolytic changes can cause mitochondrial dysfunction^11^, we analysed mitochondrial membrane potential (ΔΨm) and mitochondrial reactive oxygen species (ROS) production (MitoSOX) in BMDMs after knockdown of *PKM2*, *LDHA* or *SLC2A1* by shRNA. Indeed, knockdown of these glycolytic genes increased ΔΨm loss (Fig. 3i) and MitoSOX production (Fig. 3j) following treatment with ATP and poly (dA:dT) in LPS-primed BMDMs. Mitochondrial ROS serves as NLRP3 inflammasome activating signals^12^. Although mitochondrial ROS production is increased, the inflammation activation is inhibited in *PKM2* knockdown cells (Figs 1 and 2). These findings suggest that the PKM2-dependent glycolysis pathway is required for mitochondrial ROS-mediated inflammasome activation. Besides ROS, recent findings have demonstrated ROS-independent NLRP3 inflammasome activation^13^,^14^.

In addition to metabolic disruption, induction of autophagy by mitochondrial injury can further inhibit inflammasome activation^15^. 2DG and knockdown of *PKM2* have been demonstrated to induce autophagy by inhibition of glycolysis in cancer cells^16^,^17^. Consistently, 2DG and knockdown of *PKM2* increased microtubule associated protein 1 light chain 3 beta-II (MAP1LC3B-II, also termed LC3-II, an autophagosomal marker) expression and sequestosome 1 (SQSTM1, also termed p62, a selective substrate of autophagy) degradation in LPS-primed BMDMs after treatment with ATP or poly(dA:dT) (Fig. 3k). These findings suggest that autophagy inhibition is associated with inflammasome activation in PKM2-mediated glycolysis.

### PKM2 regulates glycolysis in macrophages

**Figure 4:**
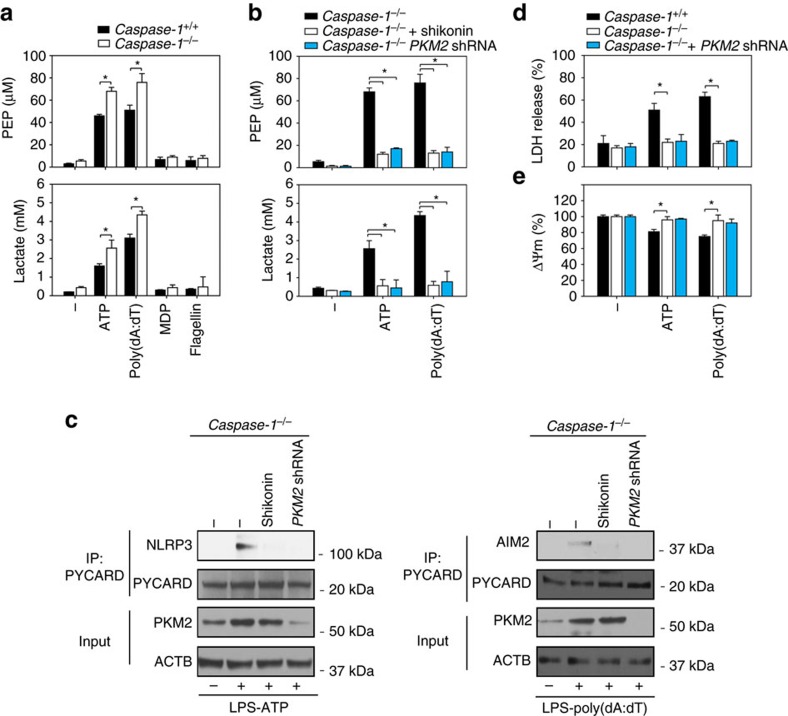
PKM2 contributes to glycolysis in caspase-1-deficient macrophages. (**a**) LPS (500 ng ml^*−*1^, 3 h)-primed WT (*caspase-1*^*+/+*^) and *caspase-1*^*−/−*^ BMDMs were treated with inflammasome activators (ATP (5 mM, 30 min), poly(dA:dT) (1 μg ml^*−*1^, 8 h), MDP (200 ng ml^*−*1^, 8 h) or flagellin (200 ng ml^*−*1^, 8 h)). The levels of PEP and lactate were assayed using a commercial kit (*n*=3, *P<0.05, ANOVA LSD test). (**b**) Inhibition of PKM2 using shikonin (5 μM) or shRNA significantly inhibited ATP- (5 mM, 30 min) or poly(dA:dT) (1 μg ml^*−*1^, 8 h)-induced PEP and lactate production in LPS (500 ng ml^*−*1^, 3 h)-primed *caspase-1*^*−/−*^ BMDMs (*n*=3, *P<0.05, ANOVA LSD test). (**c**) In parallel, ATP-induced interaction between NLRP3 and PYCARD and poly(dA:dT)-induced interaction between AIM2 and PYCARD were assayed with immunoprecipitation (IP). (**d**,**e**) Knockdown of PKM2 did not increase LDH release and ΔΨm loss in LPS (500 ng ml^*−*1^, 3 h)-primed *caspase-1*^*−/−*^ BMDMs following treatment with ATP (5 mM, 30 min) or poly(dA:dT) (1 μg ml^*−*1^, 8 h) (*n*=3, *P<0.05, ANOVA LSD test). All quantification data expressed as means±s.e.m of three independent experiments. Western blot data are representative of two independent experiments.

As an adaptive response in infection, activation of caspase-1 by NLRP3 inflammasome activators (for example, LPS+ATP) in turn reduce the cellular glycolytic rate by promoting cleavage of glycolysis enzymes such as PKM2 (ref. 18). Consistently, caspase-1 depletion increased PEP and lactate production in response to ATP or poly(dA:dT) (but not MDP or flagellin) in LPS-primed BMDMs (Fig. 4a). In contrast, inhibition of PKM2 by shikonin or shRNA significantly inhibited ATP- or poly(dA:dT)-induced PEP and lactate production in LPS-primed *caspase-1*^*−/−*^ BMDMs (Fig. 4b). Notably, ATP-induced interaction between NLRP3 and PYD and CARD domain containing (PYCARD, also termed ASC) and poly(dA:dT)-induced interaction between AIM2 and PYCARD were inhibited after knockdown of *PKM2* by shRNA in LPS-primed *caspase-1*^*−/−*^ BMDMs (Fig. 4c). We next assayed LDH release and mitochondrial ΔΨm loss in *caspase-1*^*−/−*^ BMDMs with or without PKM2 inhibition. Consistent with a previous study^11^, loss of caspase-1 inhibited ATP- or poly(dA:dT)-induced LDH release (Fig. 4d) and ΔΨm loss (Fig. 4e) in LPS-primed *caspase-1*^*−/−*^ BMDMs. However, inhibition of *PKM2* by shRNA did not affect these processes in LPS-primed *caspase-1*^*−/−*^ BMDMs (Fig. 4d,e). These data suggest that PKM2 is required for glycolysis production and PYCARD pyroptosome formation in *caspase-1*^*−/−*^ macrophages.

### Lactate promotes EIF2AK2 phosphorylation in macrophages

**Figure 5:**
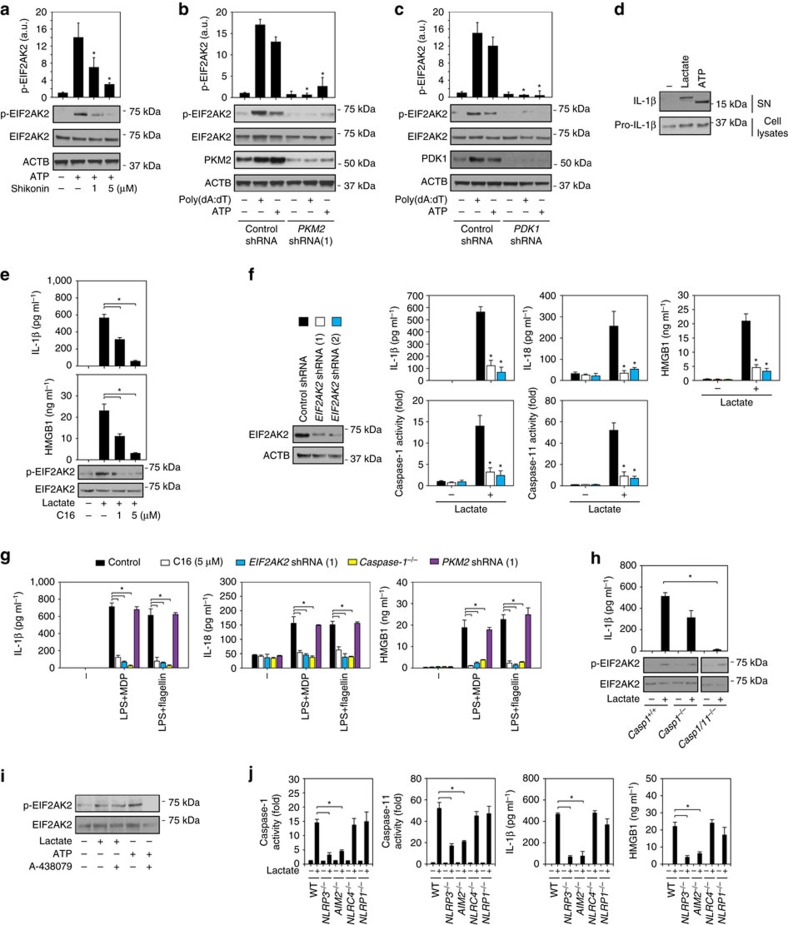
PKM2-dependent glycolysis promotes EIF2AK2 phosphorylation. (**a**) LPS-primed BMDMs were treated with ATP (5 mM, 30 min) in the absence or presence of shikonin (1 and 5 μM). p-EIF2AK2 was assayed (*n*=3, *P<0.05 versus ATP group, ANOVA LSD test). (**b**,**c**) Knockdown of *PKM2* (**b**) and *PDK1* (**c**) by shRNA inhibited ATP- (5 mM, 30 min) and poly(dA:dT) (1 μg ml^*−*1^, 8 h)-induced p-EIF2AK2 in LPS (500 ng ml^*−*1^, 3 h)-primed BMDMs (*n*=3, *P<0.05 versus control shRNA group, ANOVA LSD test). (**d**) Western blot analysis of IL-1β in culture supernatants (SN) and the precursors of IL-1β (pro-IL-1β) in lysates of LPS-primed BMDMs following treatment with lactate (5 mM, 3 h) and ATP (5 mM, 30 min). (**e**) LPS-primed BMDMs were treated with lactate (5 mM, 3 h) in the absence or presence of C16 (1 and 5 μM). IL-1β and HMGB1 release and p-EIF2AK2 expression were assayed (*n*=3, *P<0.05 versus ATP group, ANOVA LSD test). (**f**) Knockdown of *EIF2AK2* by shRNA inhibited lactate (5 mM, 3 h)-induced IL-1β, IL-18 and HMGB1 release and caspase-1/11 activation in LPS-primed BMDMs (*n*=3, *P<0.05 versus control shRNA group, ANOVA LSD test). (**g**) Inhibition of EIF2AK2 by C16 or shRNA or knockout of *caspase-1* limited MDP (200 ng ml^*−*1^, 8 h) or flagellin (200 ng ml^*−*1^, 8 h))-induced cytokine release in LPS-primed BMDMs (*n*=3, *P<0.05, ANOVA LSD test). (**h**) Double knockout of *caspase-1* and *caspase-11* inhibited lactate (5 mM, 3 h)-induced IL-1β release in LPS-primed BMDMs (*n*=3, *P<0.05, ANOVA LSD test). (**i**) A-438079 (1 μm) suppressed ATP (5 mM, 30 min), but not lactate (5 mM, 3 h)-induced p-EIF2AK2 expression in LPS-primed BMDMs. (**j**) Knockout of *NLRP3* and *AIM2* inhibited lactate (5 mM, 3 h)-induced IL-1β and HMGB1 release and caspase-1/11 activation in LPS-primed BMDMs (*n*=3, *P<0.05, ANOVA LSD test). All quantification data expressed as means±s.e.m of three independent experiments. Western blot data are representative of two independent experiments.

The phosphorylation of EIF2AK2, a protein kinase activated by viral infection, has been recently shown to be required for the activation of various inflammasomes in macrophages^7^. We next determined whether PKM2-dependent glycolysis regulates inflammasome activation by modulating EIF2AK2 phosphorylation in macrophages. Indeed, inhibition of PKM2 by shikonin (Fig. 5a) or RNAi (Fig. 5b) significantly inhibited ATP- or poly(dA:dT)-induced EIF2AK2 phosphorylation in LPS-primed BMDMs. Similarly, knockdown of PKM2-targeted glycolysis genes such as *PDK1* also inhibited ATP or poly(dA:dT)-induced EIF2AK2 phosphorylation in LPS-primed BMDMs (Fig. 5c). Lactate treatment increases IL-1β or HMGB1 release in macrophages and microglia cells^9^,^19^. Lactate was solubilized in medium without adjustment of pH. We found that the extracellular pH had changed from 7.4 to 6.6 after the addition of lactate in LPS-primed BMDMs. Similar to a previous study^20^, lactate produced a different IL-1β cleavage pattern compared with ATP treatment in LPS-primed BMDMs (Fig. 5d).

C16, a potent inhibitor of EIF2AK2 kinase activity^21^, dose-dependently inhibited lactate-induced EIF2AK2 phosphorylation and IL-1β and HMGB1 release in LPS-primed BMDMs (Fig. 5e). The knockdown of *EIF2AK2* by specific shRNA also inhibited lactate-induced IL-1β and HMGB1 release in LPS-primed BMDMs (Fig. 5f), indicating that PKM2-dependent lactate production promotes EIF2AK2 phosphorylation in inflammasome activation. Consistently with a previous study^7^, inhibition of EIF2AK2 by C16 and shRNA limited MDP- or flagellin-induced IL-1β, IL-18 and HMGB1 release (Fig. 5g), suggesting that non-PKM2-dependent upstream signalling is required for EIF2AK2-mediated NLRC4 or NLRP1 inflammasome activation in response to MDP and flagellin.

Pharmacological inhibition of caspase-1 by Z-YVAD-FMK cannot block lactate-induced inflammasome activation in glial cells^20^. We therefore addressed whether caspase-1 is also not required for lactate-induced inflammasome activation in BMDMs. However, knockout of *caspase-1* partly inhibited lactate-induced IL-1β release in LPS-primed BMDMs (Fig. 5h). In contrast, double knockout of *caspase-1* and *caspase-11* completely blocked lactate-induced IL-1β release in LPS-primed BMDMs (Fig. 5h). These findings suggest that both caspase-1 and caspase-11 (especially caspase-11) contribute to exogenous lactate-induced IL-1β release in BMDMs. Knockout of *caspase-1* or double knockout of caspase-1/11 did not affect lactate-induced EIF2AK2 phosphorylation in LPS-primed BMDMs (Fig. 5h). In contrast, knockdown of *EIF2AK2* suppressed both caspase-11 and caspase-1 activation in LPS-primed BMDMs following lactate treatment (Fig. 5f). Moreover, A-438079 (a purinergic receptor P2X 7 (P2RX7) antagonist) suppressed ATP (but not lactate)-induced EIF2AK2 phosphorylation in LPS-primed BMDMs (Fig. 5i). These findings suggest that lactate-induced EIF2AK2 phosphorylation acts upstream of caspase-1/11 activation, which is not P2RX7-dependent.

To further investigate which inflammasome is responsible for the activation of caspase-1/11 and cytokine release after lactate treatment, we treated *NLRP3*^*−/−*^, *AIM2*^*−/−*^, *NLRC4*^*−/−*^ and *NLRP1*^*−*/*−*^ LPS-primed BMDMs with lactate. Interestingly, loss of *NLRP3* and *AIM2* (but not *NLRC4* and *NLRP1*) partly limited lactate-induced caspase-1/11 activity and cytokine (IL-1β and HMGB1) release in LPS-primed BMDMs (Fig. 5j). These findings indicate that both NLRP3 and AIM2 contribute to lactate-induced inflammasome activation in LPS-primed BMDMs.

### Inhibition of the PKM2–EIF2AK2 pathway reduces septic death

**Figure 6:**
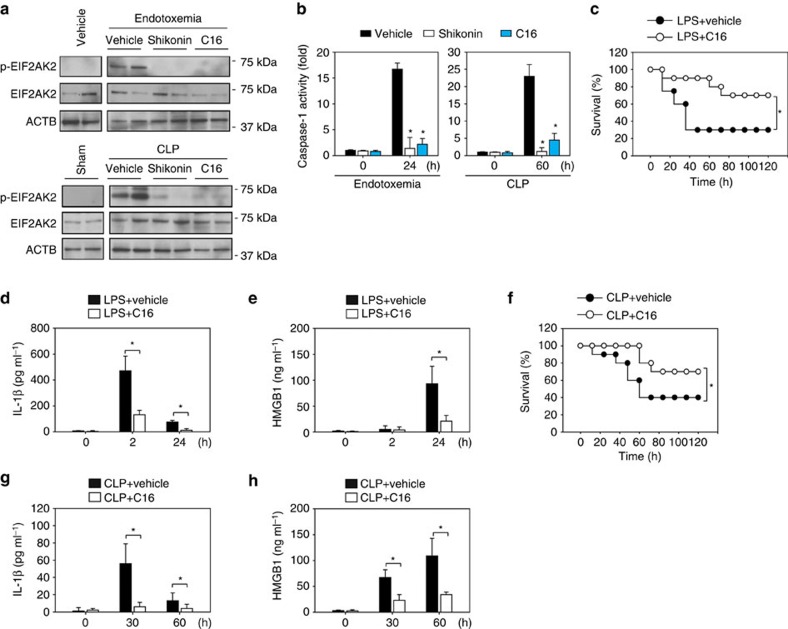
Pharmacological inhibition of the PKM2 pathway protects septic mice. (**a**,**b**) p-EIF2AK2, EIF2AK2 and caspase-1 activity were assayed in isolated PMs from mice during endotoxemia or polymicrobial sepsis in the absence or presence of shikonin (8 mg kg^*−*1^) or C16 (50 μg kg^*−*1^). In addition, the protein levels of p-EIF2AK2 and EIF2AK2 were assayed in PMs from mice with vehicle (no LPS) injection or sham operated for CLP. (**c**) Mice (*n*=20 mice per group) were injected with a single dose of C16 (8 mg kg^*−*1^), followed 30 min later by an infusion of endotoxin (LPS, 5 mg kg^*−*1^, intraperitoneally), and were then re-treated with C16 12 and 24 h later. The Kaplan–Meyer method was used to compare differences in survival rates between groups (*P<0.05). (**d**,**e**) In parallel experiments, serum levels of IL-1β and HMGB1 at indicated time points were measured (*n*=3 animals per group, *P<0.05, *t* test). (**f**) CLP was used to induce intraabdominal sepsis in mice (*n*=20 group per group). Repeated administration of C16 (50 μg kg^*−*1^) at 24, 48 and 72 h after CLP significantly increased survival compared with vehicle group (*P<0.05), as measured by Kaplan–Meyer test. (**g**,**h**) In parallel, the serum levels of IL-1β and HMGB1 at indicated time points were measured using ELISA (*n*=3 animals per group, *P<0.05, *t*-test). All quantification data expressed as means±s.e.m of three independent experiments. Western blot data are representative of two animals per group.

In light of the pathogenic role of HMGB1 in lethal sepsis, we explored the therapeutic potential of targeting the PKM2–EIF2AK2 pathway with drugs in animal models of lethal endotoxemia and polymicrobial sepsis induced by caecal ligation and puncture (CLP). Our previous study demonstrated that the PKM2 inhibitor shikonin inhibits PKM2 activity and suppresses circulating IL-1β and HMGB1 levels and prevents lethality during endotoxemia and polymicrobial sepsis in mice^9^. To gain insight into shikonin's protective mechanism, we analysed EIF2AK2 phosphorylation and caspase-1 activity in macrophages isolated from septic mice. Shikonin inhibited EIF2AK2 phosphorylation (Fig. 6a) and caspase-1 activity (Fig. 6b) in PMs obtained from mice subjected to lethal endotoxemia or polymicrobial sepsis. Similar to shikonin^9^, repetitive administration of C16 at −0.5, +12 and +24 h following the onset of endotoxemia (5 mg kg^*−*1^ LPS, i.p.) conferred significant protection against lethality (Fig. 6c) and reduced EIF2AK2 phosphorylation (Fig. 6a), and caspase-1 activity (Fig. 6b) in PMs. C16 treatment also prevented LPS-induced increases in circulating levels of IL-1β (Fig. 6d) and HMGB1 (Fig. 6e). Furthermore, C16 rescued mice from CLP-induced lethal sepsis, even if given after the onset of sepsis (Fig. 6f). EIF2AK2 phosphorylation (Fig. 6a) and caspase-1 activity (Fig. 6b) in PMs, as well as serum levels of IL-1β (Fig. 6g) and HMGB1 (Fig. 6h) were significantly lower in the group receiving C16 treatment for CLP compared with CLP alone. Collectively, these findings suggest that pharmacologic inhibition of the PKM2–EIF2AK2 pathway attenuates inflammasome activation and protects mice from lethal endotoxemia and polymicrobial sepsis.

### Knockout of PKM2 in myeloid cells reduces septic death

**Figure 7:**
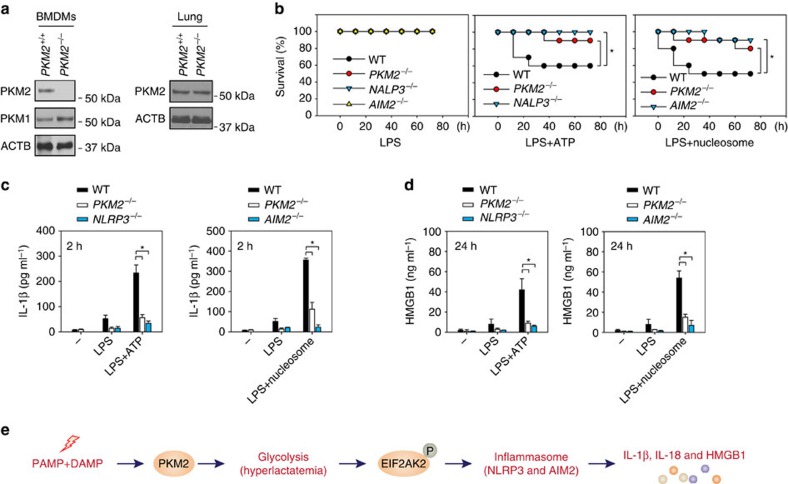
Conditional knockout of *PKM2* in myeloid cells protects septic mice. (**a**) Western blot analysis of expression of indicated proteins in BMDMs or lung isolated from myeloid cell-specific *PKM2*-knockout mice (*PKM2*^*−/−*^) and control WT mice (*PKM2*^*+/+*^). (**b**) Indicated mice (*n*=10 mice per group) were pre-injected with LPS (2 mg kg^*−*1^, intraperitoneally) for 3 h and then challenged with NLRP3 activator ATP (200 mg kg^*−*1^, intraperitoneally) or AIM2 activator nucleosome (20 mg kg^*−*1^, intraperitoneally). Injection with LPS (2 mg kg^*−*1^, intraperitoneally) alone in these mice was used as a control (*n*=10 mice per group). The Kaplan–Meyer method was used to compare differences in survival rates between groups (*P<0.05). (**c**,**d**) In parallel experiments, serum levels of IL-1β and HMGB1 at indicated time points were measured (*n*=3 animals per group, *P<0.05, ANOVA LSD test). (**e**) Schematic depicting PKM2-mediated glycolysis promoting NLRP3 and AIM2 inflammasome activation and proinflammatory cytokine (for example, IL-1β, IL-18 and HMGB1) release by modulating EIF2AK2 phosphorylation. All quantification data expressed as means±s.e.m of three independent experiments. Western blot data are representative of two independent experiments.

To further confirm the role of PKM2 in the regulation of NLRP3 and AIM2 inflammasome activation *in vivo*, we generated myeloid cell-specific *PKM2*-knockout mice (*PKM2*^*−/−*^) by crossing *PKM2*^*flox/flox*^ and *LysM-Cre*. *PKM2*^*flox/flox*^ mice possessing loxP sites flanking exon 10 of the *PKM2* gene. *LysM-Cre* mice have a *Cre* recombinase inserted into the lysozyme 2 gene and are useful for *Cre-lox* studies of myeloid cell lineage and the innate immune response. We and others recently demonstrated that nucleosome (histone+DNA) is an AIM2 activator in macrophages and mediates inflammation and tissue injury *in vivo*^22^,^23^. We primed mice with LPS (2 mg kg^*−*1^) for 3 h and then challenge with NLRP3 activator ATP (200 mg kg^*−*1^) or AIM2 activator nucleosome (20 mg kg^*−*1^). Consistent with findings from a previous study^24^, LPS at 2 mg kg^*−*1^ did not cause animal death in wild type (WT), *NLRP3*^*−/−*^, *AIM2*^*−/−*^ and *PKM2*^*−/−*^ mice. Similar to *NLRP3*^*−/−*^ and *AIM2*^*−/−*^ mice, *PKM2*^*−/−*^ mice (Fig. 7a) displayed increased animal survival (Fig. 7b) and reduced serum IL-1β (Fig. 7c) and HMGB1 (Fig. 7d) levels in response to LPS/ATP or LPS/nucleosome. These findings indicate that PKM2 is required for NLRP3 and AIM2 inflammasome activation *in vivo*. Additions of ATP or nucleosomes promote LPS-induced animal death and inflammatory response by activation of NLRP3 and AIM2 inflammasome.

## Discussion

Inflammatory cells such as macrophages play an important role in the defence against infection; however, excessive or sustained activation of these cells contributes to uncontrolled systemic inflammation during sepsis. Compared with the production and secretion of IL-1β in the early phase of macrophage activation, the release of HMGB1 is delayed and sustained^1^. The dynamics of active HMGB1 release make it a potential therapeutic target for sepsis based on a wider therapeutic window than the early cytokine mediators^25^. One of the established regulators of active HMGB1 release from macrophages is inflammasomes^2^. In this study, we demonstrated that the glycolytic regulator PKM2 selectively promotes inflammasome activation in macrophages through activating EIF2AK2 phosphorylation. Pharmacological inhibition of the PKM2–EIF2AK2 pathway reduced the release of both early (for example, IL-1β) and late (for example, HMGB1) proinflammatory mediators and protected animals against lethal sepsis. Thus, the current study indicates that PKM2-mediated metabolic programming and lactate production is critical for inflammasome activation in macrophages during sepsis (Fig. 7e).

The Warburg effect increased glycolysis, even in the presence of oxygen, namely aerobic glycolysis, in both cancer cells and activated innate immune cells. This change contributes to the regulation of innate immune functions and represents a novel target for inflammatory diseases. For instance, increased aerobic glycolysis contributes to the maturation of dendritic cells in response to toll-like receptor ligands^26^ and influences the differentiation of both anti-inflammatory T_reg_ cells and pro-inflammatory Th_17_ cells^27^,^28^. In addition to dendritic cells and T cells, LPS can also induce the Warburg effect in macrophages, which promotes IL-1β and HMGB1 release^8^,^9^,^29^. This process requires upregulated PKM2 expression that catalyses the final and rate-limiting reaction step of the glycolytic pathway. PKM2 also serves as a nuclear cofactor that interacts with the transcription factor hypoxia inducible factor 1 alpha subunit (HIF1A), thereby activating the genes involved in glycolysis in macrophages^8^. Thus, PKM2 represents a potential therapeutic target for inflammatory diseases.

PKM2-dependent glycolysis contributes to the regulation of IL-1β and HMGB1 release through multiple mechanisms, such as by facilitating HMGB1 acetylation, IL-1β transcription and/or inflammasome activation. HMGB1 is acetylated in nuclear localization signals, allowing nuclear-cytoplasmic HMGB1 translocation before extracellular secretion^30^. PKM2-mediated lactate production increases HMGB1 acetylation partly by inhibiting histone deacetylases^9^. Nuclear factor kappa B (NFKB) and HIF1A have been demonstrated to regulate IL-1β expression in macrophages^29^. Monomeric or dimeric PKM2 is required for LPS-induced binding of PKM2 and HIF1A to the IL-1β promoter, whereas tetramerization of PKM2 attenuates LPS-induced IL-1β production^8^. Activation of inflammasome is an important post-transcriptional event to promote IL-1β and HMGB1 release. The data presented here indicate that PKM2-dependent glycolysis promotes IL-1β and HMGB1 release through activation of NLRP3 and AIM2 inflammasomes in an EIF2AK2-dependent manner. Our findings agree with those from a recent report showing that hexokinase 1 (HK1)-dependent glycolysis regulates NLRP3, but not NLRP1 or NLRC4 inflammasome activation in macrophages^31^. HK1 is one of four HK isoenzymes that participate in a proximal step of glycolysis. Unlike PKM2, upregulation of HK1 is not required for AIM2 inflammasome activation^31^, suggesting that other HK members may contribute to PKM2-mediated inflammasome activation. Indeed, HK3 is significantly upregulated in macrophages after treatment with LPS^29^. Thus, synergistic upregulation of HK1 and HK3 may regulate glycolysis in activated macrophages.

The role of EIF2AK2 in inflammasome activation remains controversial. Lu *et al.*^7^ first showed that EIF2AK2 is required for inflammasome activation, whereas He *et al.*^32^ first suggested that EIF2AK2 is not required for inflammasome activation. Now many different groups support that EIF2AK2 contributes to inflammasome activation^33^,^34^,^35^,^36^,^37^, although few studies still dispute the function of EIF2AK2 in inflammasome activation^38^. Thus, context-dependent cell signalling may play an important role in the regulation of EIF2AK2-mediated inflammasome activation.

In this study, we also confirmed the role of EIF2AK2 in activation of NLRP3 and AIM2 inflammasomes in macrophages. In addition to its anti-viral functions, EIF2AK2 plays a role in inflammation and immunity regulation^39^. EIF2AK2 physically interacts with NLRP3, NLRP1, NLRC4 or AIM2, which are each involved in inflammasome assembly and activation^33^. This interaction has been shown to be mediated by EIF2AK2 phosphorylation, and kinase-defective mutant EIF2AK2 failed to bind NLRP3 (ref. 33). In contrast, a kinase-independent role for EIF2AK2 contributes to anthrax lethal toxin-induced NLRP1 inflammasome activation^37^. We demonstrated that glycolytic metabolites, including lactate, may promote EIF2AK2 phosphorylation and subsequent inflammasome activation in LPS-primed macrophages.

Serum lactate levels have been suggested as biomarkers of organ failure and mortality in patients with sepsis, trauma and other critical illnesses^40^,^41^. The Third International Consensus Definitions for Sepsis and Septic Shock (Sepsis-3) has included hyperlactatemia within the clinical criteria for septic shock^42^. Our current data provide a direct molecular link between alterations in metabolism and the inflammatory response in sepsis by glycolysis-mediated lactate production.

Lactate contributes to the inflammation process through different mechanisms. Exogenous lactate can enhance LPS-stimulated proinflammatory cytokines expression by activation of the NFKB pathway in macrophages^43^,^44^. Our current study confirms a previous study showing that lactate-mediated extracellular acidosis is required for inflammasome activation in macrophages and monocytes^20^,^45^. Moreover, monocarboxylate transporter 4-mediated lactate export is essential for the inflammatory response by maintaining a high glycolytic rate in macrophages^46^. In contrast, administration of sodium lactate (the sodium salt of lactic acid) can neutralize lactic acid and reduce inflammasome activation and organ injury in mice with immune hepatitis^47^.

The canonical inflammasomes include NLR (for example, NLRP1, NLRP3 and NLRC4) and non-NLR (for example, AIM2) types, which are mediated by activation of caspase-1 in response to pathogen-associated molecular patterns and damage-associated molecular pattern molecules (DAMPs)^48^. In contrast, non-canonical inflammasome is triggered following the binding of intracellular LPS to caspase-11 (in mice) or caspase-4 (in humans) or caspase-5 (in humans)^49^,^50^,^51^,^52^. Activation of both caspase-1 and caspase-11 contributes to sepsis^53^,^54^. Interplay between canonical and non-canonical inflammasomes and other types of regulated cell death may amply the inflammatory response in Gram-negative bacterial infection^55^,^56^. Our study indicates a sophisticated interplay between caspase-1 and caspase-11 that connects the canonical and noncanonical pathways of inflammasome activation in response to lactate.

In addition, our data point to the therapeutic potential of shikonin and C16 in the setting of sepsis. Shikonin is a major component of zicao (purple gromwell, the dried root of *Lithospermum erythrorhizon*), a Chinese herbal medicine with anti-inflammatory properties^57^. C16 has been shown to effectively inhibit EIF2AK2 function and protect against neuroinjury and neuroinflammation in animal studies^58^. We demonstrated that both shikonin and C16 protect mice from lethal endotoxemia and polymicrobial sepsis in association with suppressed inflammasome activation. Consistently, conditional knockout of *PKM2* in myeloid cells in mice protects against death from activation of NLRP3 and AIM2 inflammasome by ATP and nucleosome, respectively. ATP plays dual roles in death and survival. As a DAMP, ATP release by dead or dying cells can trigger NLRP3 inflammasome activation and proinflammatory release in bacterial infection. In contrast, ATP can enhance phagocytosis in macrophages to clear bacteria in sepsis^59^. In addition to ATP, significantly higher nucleosome (histone and DNA) levels have been reported in patients with systemic inflammation and sepsis. As a DAMP, nucleosome is a mediator of death in sepsis and pancreatitis^60^,^61^. Thus, blocking DAMP release may limit inflammasome activation in infection.

In summary, we demonstrate here that PKM2 plays a critical role not only in metabolic reprogramming, but also in inflammasome activation and subsequent proinflammatory mediator release in macrophages. We also demonstrated that lactate-mediated EIF2AK2 phosphorylation is a major event that controls inflammasome activation in macrophages. It will be of interest to assess whether pharmacological modulation of the PKM2-EIF2AK2 pathway confers protection in other models of local and systemic inflammation.

## Methods

### Reagents

LPS (*Escherichia coli* LPS 0111:B4; #L4391), 2DG (#D8375), C16 (#I9785), A438079 (#A9736) and lactate (lactic acid, #L6661) were obtained from Sigma (St Louis, MO, USA). Shikonin (#CAS 517-89-5) was obtained from Millipore Corporation (Billerica, MA, USA). ATP (#tlrl-atp), poly(dA:dT)/LyoVec (#tlrl-patc), LyoVec (#lyec-12), MDP (#tlrl-mdp) and flagellin (#tlrl-stfla) were obtained from InvivoGen (San Diego, CA, USA). Purified nucleosome (#16-0002) was obtained from EpiCypher (Research Triangle Park, NC, USA)

### Cell culture

Mouse PMs and BMDMs were isolated from Balb/C mice as previously described^62^,^63^. Immortalized BMDMs from WT, *NLRP3*^*−/−*^, *AIM2*^*−/−*^, *NLRC4*^*−/−*^, *NLRP1*^*−/−*^, *caspase-1*^*−/−*^ and *caspase-1/11*^*−/−*^ mice were a kind gift from Dr Kate Fitzgerald (University of Massachusetts Medical School, Worcester, MA) and Dr Eicke Latz (University of Bonn, Bonn, Germany). THP1 cells were obtained from American Type Culture Collection (#TIB-202, Manassas, VA, USA). These cells were cultured in Dulbecco's Modified Eagle's Medium or RPMI-1640 Medium (supplemented with 10% heat-inactivated fetal bovine serum and 100 units of penicillin and 100 μg ml^*−*1^ streptomycin) at 37 °C, 95% humidity and 5% CO_2_. THP1 cells were differentiated into macrophages in RPMI-1640 medium containing PMA (5 ng ml^*−*1^) over 48 h (ref. 64). BMDMs, PMs and PMA-differentiated THP1 were primed with LPS (500 ng ml^*−*1^) for 3 h, followed by stimulation of inflammasome activators: ATP (5 mM, 30 min), poly(dA:dT) (1 μg ml^*−*1^, 8 h), MDP (200 ng ml^*−*1^, 8 h) or flagellin (200 ng ml^*−*1^, 8 h). All cells were mycoplasma free and authenticated by Short Tandem Repeat DNA Profiling Analysis.

### Animal model of endotoxemia and sepsis

Endotoxemia was induced in Balb/C mice (male and female, 7–8 weeks old, 20–25 g) by intraperitoneal (i.p.) injection of bacterial endotoxin (LPS, 5 mg kg^*−*1^)^1^,^65^. Sepsis was induced in Balb/C mice (male and female, 7–8 weeks old, 20–25 g) by caecal ligation and puncture^1^,^65^. Mice were randomly allocated into treatment groups and no blinding was done. Shikonin and C16 were dissolved in vehicle (10% DMSO, 20% cremophor:ethanol [3:1] and 70% phosphate-buffered saline) and administered i.p. to mice at the indicated time points. Blood was collected at indicated time points, allowed to clot for 2 h at room temperature, and then centrifuged for 15 min at 1,500*g*. Serum samples were stored at −20 °C before analysis. Mortality was recorded for up to 2–3 weeks after the onset of lethal endotoxemia or sepsis to ensure that no additional late deaths occurred.

Myeloid cell-specific *PKM2*-knockout mice were bred by crossing *PKM2*^*flox/flox*^ (strain name: B6;129S-Pkm^tm1.1Mgvh/J^; #024048; The Jackson Laboratory) and *LysM-Cre* (strain name: B6.129P2-Lyz2^tm1(cre)Ifo/J^; #004781; The Jackson Laboratory) transgenic mice.

We conducted all animal care and experimentation in accordance with the Association for Assessment and Accreditation of Laboratory Animal Care guidelines ( http://www.aaalac.org/) and with approval from the Institutional Animal Care and Use Committees from the Xiangya Hospital, University of Pittsburgh, or Third Military Medical University.

### Cytokine measurements

Commercially available enzyme-linked immunosorbent assay (ELISA) kits were used to measure the concentrations of HMGB1 (Shino Test Corporation, Tokyo, Japan, #ST51011), IL-1β (R&D, # MLB00C and #DY201) and IL-18 (R&D, #7625, and #7620) in serum or culture medium according to the manufacturer's instructions.

### Biochemical assays

Lactate in culture medium was measured with the L-Lactate Assay Kit (Abcam, Cambridge, MA, USA, #ab65331) according to the manufacturer's instructions. PEP production in cell lysates was measured using a PEP Assay Kit (Cayman Chemical, Ann Arbor, Michigan, USA, #700780) according to the manufacturer's instructions. Relative activity or expression of caspase-1 or caspase-11 in cell lysates was measured with the caspase-1 Assay Kit (Abcam, Cambridge, MA, USA, #ab39412) or caspase-11 Assay Kit (Abbexa Ltd, Cambridge, United Kingdom, #abx255239) according to the manufacturer's instructions. LDH release in culture medium was measured with the LDH Assay Kit (Abcam, Cambridge, MA, USA, # ab102526) according to the manufacturer's instructions.

### ΔΨm and MitoSox

Cells were seeded in 96-well plates and cultured in the presence of a given stimuli for the indicated time. Mitochondrial changes in mitochondrial membrane potential (ΔΨm) in live cells was measured with the TMRE-Mitochondrial Membrane Potential Assay Kit (Abcam, Cambridge, MA, USA, #ab113852) according to the manufacturer's instructions. Mitochondrial superoxide was assessed with the cell permeable dye MitoSox (Thermo Fisher Scientific Inc., #M36008) according to the manufacturer's instructions. Analysis of signal intensity was performed using a fluorescent plate reader.

### RNAi

All shRNA constructs were in the pLKO.1 backbone. Transfection with mouse PKM2-shRNA-1 (5′-CCGGATCATTGCCGTGACTCGAAATCTCGAGATTTCGAGTCACGGCAATGATTTTTTG-3′), mouse PKM2-shRNA-2 (5′-CCGGGATGTCGACCTTCGTGTAAACCTCGAGGTTTACACGAAGGTCGACATCTTTTTG-3′), mouse GLUT1-shRNA (5′-CCGGGCCATGAGCTTTGTCTGTATTCTCGAGAATACAGACAAAGCTCATGGCTTTTTG-3′), mouse LDHA-shRNA (5′-CCGGGTTCCCAGTTAAGTCGTATAACTCGAGTTATACGACTTAACTGGGAACTTTTTG-3′), mouse PDK1-shRNA (5′-CCGGGCTGAGTATTTCTTTCAAGTTCTCGAGAACTTGAAAGAAATACTCAGCTTTTTG-3′), mouse EIF2AK2-shRNA-1 (5′-CCGGGGAGTAGCCATTACGTATAAACTCGAGTTTATACGTAATGGCTACTCCTTTTTG-3′), mouse EIF2AK2-shRNA-2 (5′-CCGGCGCCAGGTTTAACAGCGATTTCTCGAGAAATCGCTGTTAAACCTGGCGTTTTTG-3′), human PKM2-shRNA-1 (5′-CCGGGTTCGGAGGTTTGATGAAATCCTCGAGGATTTCATCAAACCTCCGAACTTTTTTG-3′), human PKM2-shRNA-2 (5′- CCGGGCCCGAGGCTTCTTCAAGAAGCTCGAGCTTCTTGAAGAAGCCTCGGGCTTTTTTG-3′), control empty shRNA (#SHC001) from Sigma were performed using MISSION shRNA Lentiviral Transduction (Sigma) according to the manufacturer's instructions. The same shRNA was used to knock down the same genes in mouse BMDMs and PMs.

### Western blot

Proteins in cell lysates were first resolved using sodium dodecyl sulfate (SDS)-polyacrylamide gel electrophoresis, then transferred to nitrocellulose membrane, and subsequently incubated with the primary antibody as previously described^66^,^67^. The antibody to IL-1β (#AF-401-NA; 1:500) was obtained from R&D (Minneapolis, MN, USA). The antibodies to caspase-1 (#AG-20B-0042; 1:1,000) and NLRP3 (#AG-20B-0014: 1:1,000) were obtained from Adipogen (San Diego, CA, USA). The antibodies to PKM2 (#4053; 1:1,000), PKM1 (#7067; 1:1,000), ACTB (#3700; 1:3,000), AIM2 (#13095; 1:1,000), and PDK1 (#3062; 1:1,000) were obtained from Cell Signaling Technology (Danvers, MA, USA). The antibodies to EIF2AK2 (#sc-1702; 1:200), SQSTM1 (#sc-28359; 1:200), and PYCARD (#sc-22514-R; 1:200) were obtained from Santa Cruz Biotechnology (Dallas, Texas, USA). The antibody to p-EIF2AK2 (#07-886; 1:500) was obtained from Millipore (Billerica, MA, USA). The antibody to MAP1LC3B (#NB100-2220; 1:1,000) was obtained from Novus Biologicals (Littleton, CO, USA). After incubation with peroxidase-conjugated secondary antibodies, the signals were visualized using enhanced chemiluminescence (Pierce, Rockford, IL, USA, # 32106) according to the manufacturer's instructions. Native gel electrophoresis assays were performed to evaluate the formation of dimeric PKM2^68^. Full scan of blots are presented in Supplementary Fig. 3.

### Q-PCR

Total RNA was extracted using TRI reagent (Sigma) according to the manufacturer's instructions. First-strand cDNA was synthesized from 1 μg of RNA using the iScript cDNA Synthesis kit (Bio-Rad, Hercules, CA). cDNA from various cell samples were amplified by real-time quantitative PCR (Q-PCR) with specific primers (*PKM2*: 5′-TCGCATGCAGCACCTGATT-3′ and 5′-CCTCGAATAGCTGCAAGTGGTA-3′; *SLC2A1*: 5′-ATGGATCCCAGCAGCAAG-3′ and 5′-CCAGTGTTATAGCCGAACTGC-3′; *LDHA*: 5′-GCTCCCCAGAACAAGATTACAG-3′ and 5′-TCGCCCTTGAGTTTGTCTTC-3′; *PDK1*: 5′-CCACTGAGGAAGATCGACAGAC-3′ and 5′-AGAGGCGTGATATGGGCAATCC-3′; *IL-1β*: 5′-TGGACCTTCCAGGATGAGGACA-3′ and 5′-GTTCATCTCGGAGCCTGTAGTG-3′; *HMGB1*: 5′-CCAAGAAGTGCTCAGAGAGGTG-3′ and 5′-GTCCTTGAACTTCTTTTTGGTCTC-3′; *NLRP3*: 5′-TCACAACTCGCCCAAGGAGGAA-3′ and 5′-AAGAGACCACGGCAGAAGCTAG-3′; *AIM2*: 5′-AGGCTGCTACAGAAGTCTGTCC-3′ and 5′-TCAGCACCGTGACAACAAGTGG-3′) and the data was normalized to *18S ribosomal* RNA (5′-CTTAGAGGGACAAGTGGCG-3′ and 5′-ACGCTGAGCCAGTCAGTGTA-3′).

### Immunoprecipitation

Cells were lysed at 4 °C in ice-cold modified radioimmunoprecipitation lysis buffer (Millipore, Billerica, MA, USA) and cell lysates were cleared by centrifugation (12,000*g*, 10 min). Concentrations of proteins in the supernatant were determined by bicinchoninic acid assay. Before immunoprecipitation, samples containing equal amounts of proteins were pre-cleared with Protein A/G agarose/sepharose beads (Thermo Fisher Scientific Inc., #20423) (4 °C, 3 h) and subsequently incubated with various irrelevant IgG or specific antibodies (2–5 μg ml^*−*1^) in the presence of Protein A/G agarose/sepharose beads overnight at 4 °C with gentle shaking. Following incubation, agarose/sepharose beads were washed extensively with phosphate-buffered saline and proteins were eluted by boiling in 2 × SDS sample buffer before SDS–PAGE electrophoresis.

### Statistical analysis

Data are expressed as means±s.e.m of three independent experiments. Significance of differences between groups were determined using ANOVA LSD or *t*-test. The Kaplan–Meier method was used to compare the differences in mortality rates between groups. A *P* value <0.05 was considered statistically significant.

### Data availability

The authors declare that the data supporting the findings of this study are available within the article and its Supplementary information files.

## Additional information

**How to cite this article:** Xie, M. *et al.* PKM2-dependent glycolysis promotes NLRP3 and AIM2 inflammasome activation. *Nat. Commun.* **7,** 13280 doi: 10.1038/ncomms13280 (2016).

## Supplementary Material

Supplementary Information Supplementary Figures 1-3
