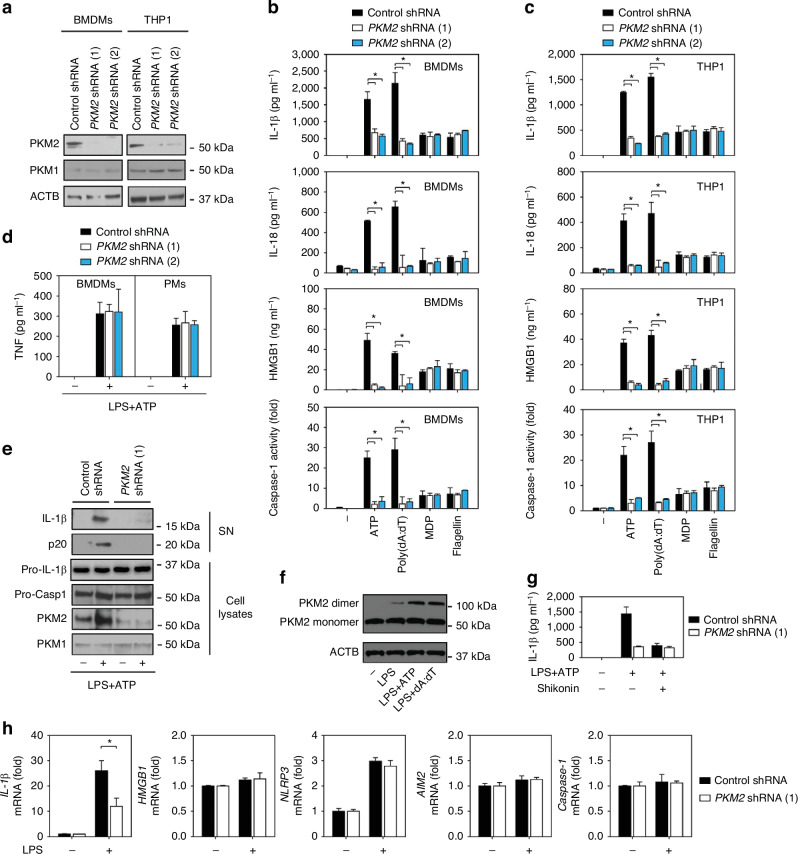# Author Correction: PKM2-dependent glycolysis promotes NLRP3 and AIM2 inflammasome activation

**DOI:** 10.1038/s41467-025-60997-7

**Published:** 2025-07-04

**Authors:** Min Xie, Yan Yu, Rui Kang, Shan Zhu, Liangchun Yang, Ling Zeng, Xiaofang Sun, Minghua Yang, Timothy R. Billiar, Haichao Wang, Lizhi Cao, Jianxin Jiang, Daolin Tang

**Affiliations:** 1 Department of Pediatrics, Xiangya Hospital, Central South University, Changsha, Hunan 410008 China; 2 Department of Surgery, University of Pittsburgh, Pittsburgh, Pennsylvania 15219 USA; 3 Center of DAMP Biology, The Third Affiliated Hospital of Guangzhou Medical University, Guangzhou, Guangdong 510510 China; 4 State Key Laboratory of Trauma, Burns and Combined Injury, Research Institute of Surgery, Research institute for Traffic Medicine of People’s Liberation Army, Daping Hospital, Third Military Medical University, Chongqing, 400042 China; 5 Laboratory of Emergency Medicine, The Feinstein Institute for Medical Research, Manhasset, New York 11030 USA

Correction to: *Nature Communications* 10.1038/ncomms13280, published online 25 October 2016

In the version of the article initially published, the western blot band for pro-IL-1β in Fig. 2e was incorrect. The correct Fig. 2 is shown below. This notice serves to correct the error.

Corrected Fig. 2