# Supplementary material for: PKM2-dependent glycolysis promotes NLRP3 and AIM2 inflammasome activation
Source: Nat Commun. 2016 Oct 25;7:13280. doi: 10.1038/ncomms13280 (PMC5093342; doi:10.1038/ncomms13280)

PKM2-dependent Glycolysis Promotes NLRP3 and AIM2 Inflammasome Activation

Supplementary Information

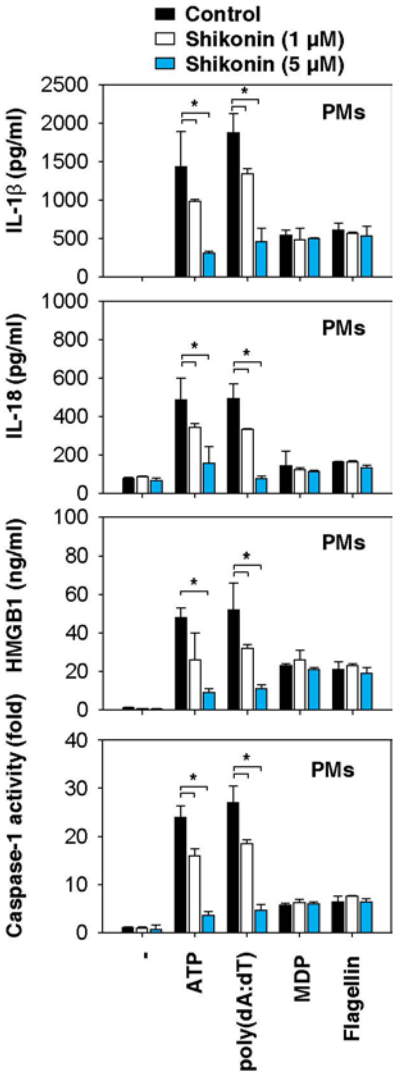

Supplementary Figure 1. Pharmacologic inhibition of PKM2-impaired NLRP3 and AIM2 inflammasome activation in macrophages. LPS (500 ng ml^{-1}, 3 hours)-primed mouse PMs were treated with inflammasome activators [ATP (5 mM, 30 min), poly(dA:dT) (1 μg ml^{-1}, eight hours), MDP (200 ng ml^{-1}, 8 hours), or flagellin (200 ng ml^{-1}, eight hours)] in the absence or presence of shikonin at same time (1 and 5μM). IL-1β, IL-18, and HMGB1 in supernatants and caspase-1 activity in whole cell extract were assayed (n=3, *P<0.05, ANOVA LSD test). All quantification data are presented as mean±s.e.m.

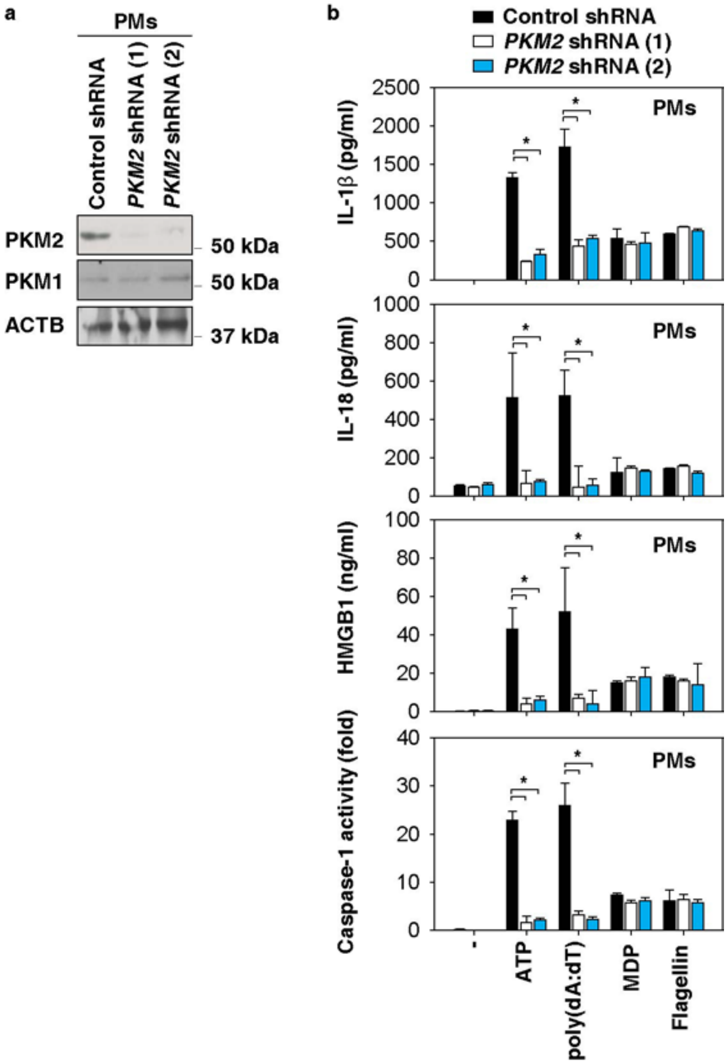

Supplementary Figure 2. Genetic inhibition of *PKM2* suppressed NLRP3 and AIM2 inflammasome activation in macrophages. (a) Western blot analysis of PKM2 expression in control and *PKM2* shRNA-treated PMs. (b) LPS (500 ng ml^{-1}, three hours)-primed PMs were treated with various inflammasome activators [ATP (5 mM, 30 min), poly(dA:dT) (1 μg ml^{-1}, eight hours), MDP (200 ng ml^{-1}, 8 hours), or flagellin (200 ng ml^{-1}, 8 hours)]. Extracellular levels of IL-1β, IL-18, and HMGB1 and cellular levels of caspase-1 were assayed (n=3, *, $P<0.05$, ANOVA LSD test). All quantification data are presented as mean±s.e.m.

Supplementary Figure 3. Full scan of blot experiments

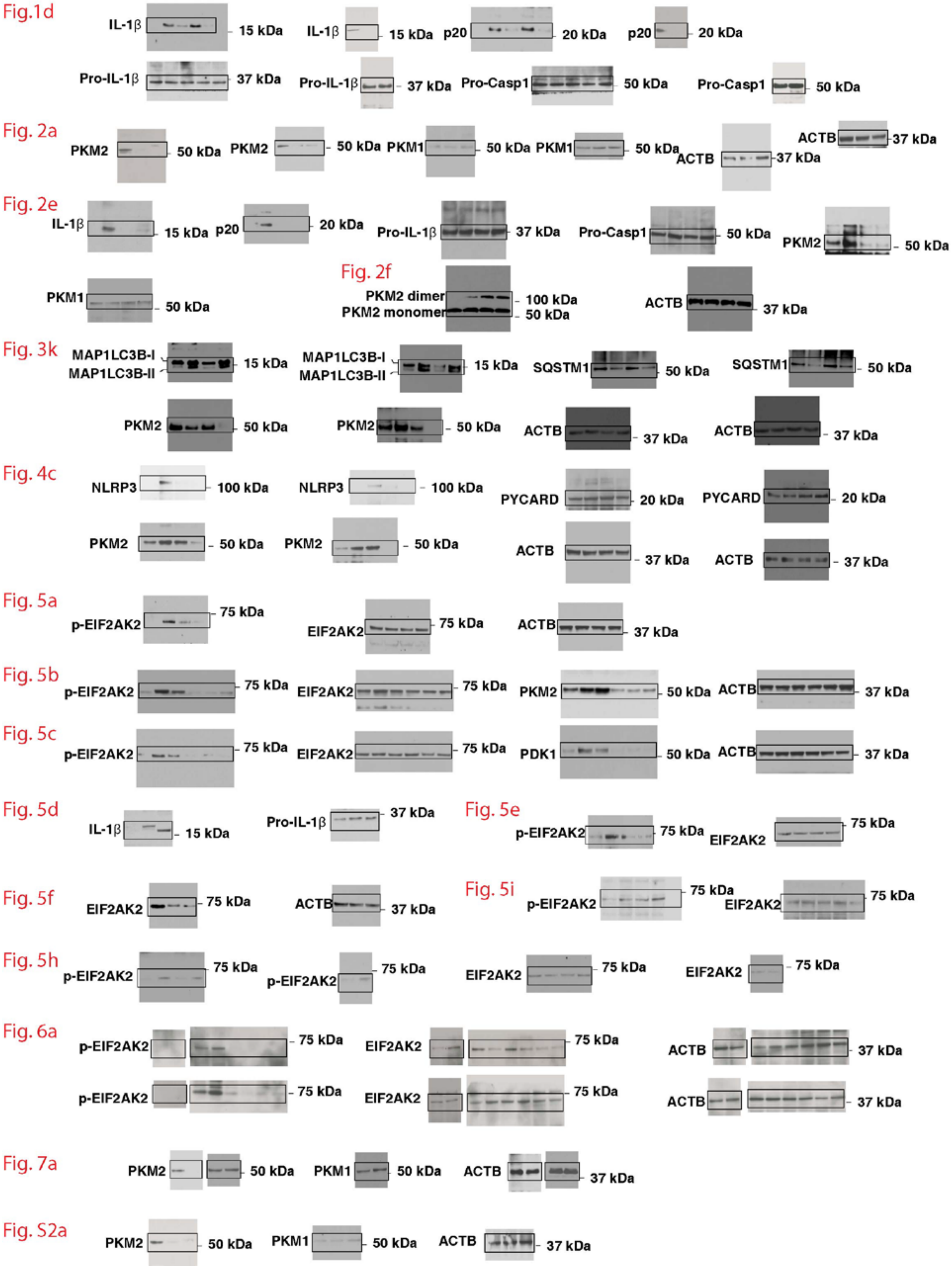

Supplement: Supplementary Information — Supplementary Figures 1-3 [file ncomms13280-s1.pdf]
